# A comparative study of conservation and variation scores

**DOI:** 10.1186/1471-2105-11-388

**Published:** 2010-07-21

**Authors:** Fredrik Johansson, Hiroyuki Toh

**Affiliations:** 1 Division of Bioinformatics, Medical Institute of Bioregulation, Kyushu University, 3-1-1 Maidashi, Higashi-ku, Fukuoka 812-8582, Japan; 2 CBRC, AIST Tokyo Waterfront Bio-IT Research Building, 2-42 Aomi, Koto-ku, Tokyo 135-0064, Japan

## Abstract

**Background:**

Conservation and variation scores are used when evaluating sites in a multiple sequence alignment, in order to identify residues critical for structure or function. A variety of scores are available today but it is not clear how different scores relate to each other.

**Results:**

We applied 25 conservation and variation scores to alignments from the Catalytic Site Atlas (CSA). We calculated distances among scores based on correlation coefficients, and constructed a dendrogram of the scores by average linking cluster analysis. The cluster analysis showed that most scores fall into one of two groups--substitution matrix based group and frequency based group respectively. We also evaluated the scores' performance in predicting catalytic sites and found that frequency based scores generally perform best.

**Conclusions:**

Conservation and variation scores can be classified into mainly two large groups. When using a score to predict catalytic sites, frequency based scores that also consider a background distribution are most successful.

## Background

A protein's amino acid sequence is considered to carry information about structure and function of the protein. However, it is still difficult to predict residues important for structure and function from a single sequence. One effective way of extracting such information is the comparison of homologous sequences, since proteins sharing a common ancestry often are similar in structure and function. Therefore, the residues critical for function or structure have been conserved in homologous proteins during the course of molecular evolution. In other words, we can predict the residues or alignment sites under strong constraints by comparing amino acid sequences of homologous proteins and identifying conserved alignment sites. Not only conservation, but also variability sometimes provides important information about proteins. As an example, consider a viral peptide antigen, which is a target for the immune system of the hosts. The amino acid residue at a site recognized by the immune system of hosts would change rapidly to escape an attack by the immune system. Therefore, the antigenicity-determining sites can be predicted by evaluating the variability of alignment sites.

A quantitative measure for conservation or variability of alignment sites is useful for identifying sites under constraints, and various methods to quantitatively evaluate the conservation or variability of alignment sites have been developed. The methods are hereafter referred to as scoring methods or simply as scores. Such scores have been reviewed and classified based on calculation method by Valdar [1], although new scores have been developed since then. There is however yet no report which systematically examines the practical similarities or performances of scoring methods. Such information would be useful when several scoring methods are available to analyze a multiple sequence alignment.

We present here an empirical comparison of scoring methods. We have collected programs for scoring methods--some were implemented by ourselves and others were provided by the developers. We apply the methods to a subset of the Catalytic Site Atlas [2], which is a dataset containing alignments as well as information about catalytic sites. We calculate a distance matrix using the correlation coefficients between scoring methods and perform a cluster analysis on the scoring methods. We also evaluate the scores' performance in predicting catalytic sites, which are sites under strong evolutionary constraints.

## Results

One simple way of evaluating the similarity between a pair of scores is to calculate a correlation coefficient between the two scores over alignment sites in a whole dataset. However, if one score is highly affected by the number of sequences in one alignment, this simple correlation does not reflect actual similarity. We therefore first examine the dependency of each score on alignments size. Strictly speaking, we should consider effective alignment size by taking sequence weights into account. We however take a simpler way of using the number of sequences as measure of alignment size since some methods do not use sequence weights and are therefore considered to depend on alignment size directly.

The correlations between alignment size *N *and mean score is shown in Table 1, and we can see that there is a variety of correlations, ranging from highly negative to highly positive. The majority of scores show a negative correlation, which may be explained in part by a possible higher sequence diversity for higher *N*. This can however not explain the positive correlations. The positive correlation of Lockless99 is explained by the fact that it calculates binomial probabilities for the number of occurences of amino acids. The probabilities for *n_k _*become very small for increasing values of *N*, giving a higher conservation score (the denominator in the definition of Lockless99 is not affected as much, since the average occurence *n *generally does not deviate much from the expected occurence *qN*).

**Table 1:** Pearson correlation of alignment size (*N*) and mean conservation score on the CSA dataset.

0.97	Lockless99	-0.42	Pei01varw
0.51	Mayrose04	-0.50	Shannon
0.02	Sander91sp	-0.55	Caffrey04w
-0.08	Valdar01	-0.55	Wang06w
-0.11	Karlin96	-0.56	Shannonw
-0.18	Thompson97	-0.64	Mihalek07
-0.20	Liu08w	-0.66	Capra07w
-0.26	Pei01sp	-0.71	Zvelebil87
-0.31	Pei01spw	-0.74	Wu70
-0.33	Mirny99	-0.75	Taylor86
-0.36	Pei01var	-0.92	Zhang08
-0.39	Williamson95	-0.92	Mihalek04

Variation scores have been negated into conservation scores.

It is not straightforward to exclude the dependencies found. For example, we divided the real-valued evolutionary trace Mihalek04 by *N *- 1 (since it is a summation of *N *- 1 terms) which did not abolish the dependency on alignment size (data not shown). The conclusion will have to be that a comparison of scores in different alignments should not be done, and we therefore adopt the procedure of calculating correlations only within alignments as described in Methods for the cluster analysis.

### Clustering results

**Figure 1:**
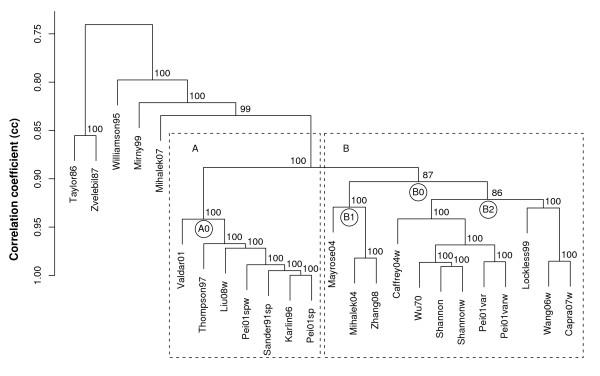
**Hierarchical clustering results**. Dendrogram obtained from hierarchical clustering of average Spearman correlations on each alignment, using average linking. Each node is labeled by a probability value in percent, given by 1000 iterations of a bootstrap procedure.

The result of the cluster analysis using average linking is shown in the dendrogram in Figure 1. Each node in Figure 1 shows where scoring methods under it are joined at an average correlation coefficient (*acc*). In cases where only two scoring methods are joined, we refer to it as simply the correlation coefficient (*cc*). Each node is labeled with the result from a bootstrap analysis, where the number is to be interpreted as the percentage of bootstrap samples that have an identical node. Generally, the dendrogram was found to be very stable by the bootstrap procedure, as can be seen by the fact that many nodes have been labeled with 100. We define two major groups (A and B) which are illustrated by squares in the dendrogram. They are clustered with bootstrap probabilities of 100% and 87% respectively.

#### Cluster A

The top node in cluster A is labeled as A0 in Figure 1 and is found at *acc *= 0.94 with a bootstrap probability of 100%. The internal topology of cluster A is also very stable, as can be seen on the bootstrap probabilities on the nodes internal to this cluster. This cluster holds all the scores described above as "substitution matrix scores" (see Methods), except for Mihalek07.

The highest correlations in cluster A are found between Karlin96, Pei01sp(w) and Sander91sp, which are all clustered at *acc *≥ 0.99. The scoring methods Karlin96 and Sander91sp are very similar already in their formulations, but we can also conclude that Pei01sp(w) is very similar in practice even though the score formulation is quite different. Hence, at least for this dataset, the weighting of Pei01sp does not make much difference. We may also note that even though Sander91sp uses sequence weights for each pair, this does not make any practical difference compared to Karlin96 and Pei01sp that do not use sequence weights at all. All these scores also use the same matrix *M_K _*in their calculations, which may explain their similarities. The other scores in cluster A are Valdar01, Thompson97 and Liu08w. Valdar01 is by its definition quite similar to Karlin96 and Sander91sp, using different sequence weighting and a different scoring matrix. Thompson97 measures the deviation from a hypothetical "consensus residue". It measures this deviation using a scoring matrix just as the other scores in cluster A, which is seen to give Thompson97 a very similar behaviour. Liu08w compares each occuring amino acid to the most common amino acid at the site, also using a similar scoring matrix.

#### Cluster B

A variety of scores are classified into cluster B, which is formed at node B0 located at *acc *= 0.90 in the dendrogram in Figure 1. However, all scores in this cluster share the property that they consider individual amino acids rather than amino acid substitution as the scores in cluster A do. Cluster B is divided at node B0 into phylogeny- and non-phylogeny based scores, labeled by B1 and B2 respectively in Figure 1. Subgroup B1 contains all scores that use a phylogenetic tree for their analyses. There is a very close relation (*cc *= 0.98) between Mihalek04 and Zhang08, and they do indeed share a summation over the same phylogenetic tree. Zhang08 uses von Neumann entropy, which considers the amino acid similarities that the Shannon entropy used by Mihalek04 does not. The summation over the same phylogenetic tree seems to erase most differences between these entropies, however. We may compare this pair to their phylogeny-free counterparts Shannonw and Caffrey04w (see Subgroup B2), which show a slightly larger difference (*cc *= 0.96).

Mayrose04 is the most distant relative in cluster B, even though its closest relatives are the other phylogeny-based scores. Mayrose04 is a computationally different method, but its score is also conceptually different. It uses mutation probabilities from the JTT matrix [3], and calculates evolutionary rates given these probabilities. This has the effect of estimating a lower evolutionary rate (hence higher conservation score) for the conservation of an amino acid that typically mutates easily (e.g. Ala) than for an amino acid that typically does not mutate easily (e.g. Trp). This is in stark contrast to the relative entropy scores Wang06w and Capra07w, considering the fact that rare amino acids are often also amino acids that do not mutate easily.

Subgroup B2 contains scores that do not consider phylogeny in their scoring. This subgroup is vaguely divided into scores that do or do not consider background distributions, where the scores that do consider a background are located to the right of node B2. The exception to this rule is Pei01var and Pei01varw. By inspecting the correlation matrix, we could however see that Pei01var had a quite high correlation (*cc *= 0.97) with Lockless99 while correlating with *cc *= 0.98 to Shannon. Hence, Pei01var(w) is not as unrelated to the relative scores Lockless99, Wang06w and Capra07w as it may seem by the dendrogram. Wu70 correlates with *cc *= 0.99 to both Shannon and Shannonw, and we may conclude that the formulation of Wu70 is in practice very similar to an entropy. Caffrey04w is also clustered together with these "non-relative" scores, though at some distance. Wang06w and Capra07w are clustered together at *cc *= 0.99, which is not surprising since they are very similar by their definitions. Wang06w uses relative entropy while Capra07w uses the Jensen-Shannon divergence that has relative entropy in its definition. As mentioned above, Lockless99 is clustered together with these scores which is expected since Lockless99 is a score that also measures deviation from a background.

#### Other scores

Outside the clusters described above we find scores that do not relate closely to any other score, which may be concluded from the dendrogram where all joining nodes outside clusters A and B are found at *acc *≤ 0.86. All scores that are based on a manual grouping of amino acids are found among these scores. Williamson95 measures relative entropy on an alphabet of nine amino acid groups, Mirny99 measures entropy on an alphabet of six amino acid groups, and Taylor86 and Zvelebil87 measure conservation of properties. They all have different approaches on how to group the amino acids, and this gives quite different behaviour for each of these scores.

Mihalek07 measures the relative entropy of residue pairs at an alignment site, where the background is given by the substitution matrix *M_M_*. This matrix is normalized so that each row and column sum to approximately one, and the element *M_M _*(*α*, *β*) may be interpreted as a probability that the given amino acid *α *will mutate to amino acid *β *(or be conserved if *α *= *β*). A feature of this score, which may be what makes it different from most other scores, is that rare mutations show a large relative entropy (hence a large conservation score). Mihalek *et al. *[4] argue that a rare mutation should imply an important meaning of the site.

#### Other clustering methods

Dendrograms created by single linking and complete linking are shown in Additional file 1 and 2 respectively. There are many similarities to the average linking shown in Figure 1, though there are also many differences. Nodes close to the leaves are similar using any method, which may be expected since all methods are identical if measuring distance between single units.

With any clustering method, cluster A is formed with the same topology and high statistical significance, except for Valdar01 in complete linking. This is due to the fact that Valdar01 correlates very strongly (*cc *≥ 0.95) to most scores in cluster A, except for Thompson97 to which it correlates with only *cc *= 0.91. By complete linking, Valdar01 is then put in a different cluster, where it correlates to all other scores with *cc *≥ 0.92. This illustrates a weakness of complete clustering, where the similarities between Valdar01 and other scores in cluster A are not visible in the dendrogram created by this method.

Neither single linking nor complete linking recreates cluster B. With single linking it is instead spread out in what is often referred to as the "chaining effect", meaning that links may be created without regard to the shape of the emerging cluster. However, the subcluster consisting of Pei01var, Pei01varw, Wu70, Shannon and Shanonw is found with the same topology and statistical significance by any clustering method, suggesting that symbol frequency methods except for Lockless99 are highly similar to symbol entropy methods.

Using complete linking, subcluster B1 of Figure 1 is merged with cluster A, together with Mihalek07, Mirny99 and Caffrey04w. These are all scores which consider the stereochemical similarities of amino acid, and this may be the reason why they are placed close to cluster A in this dendrogram.

Both Taylor86 and Zvelebil87 belong to the stereochemical properties group, and are clustered together by any clustering method. Since this subcluster is distantly located from other scoring methods also by single linking we can see that they are not closely related to any other score.

Thus, the results of these two cluster analyses provide supporting evidence for the classification of a cluster and some subclusters created by the average linking method, despite the difference in clustering pattern.

### Performance evaluation

**Figure 2:**
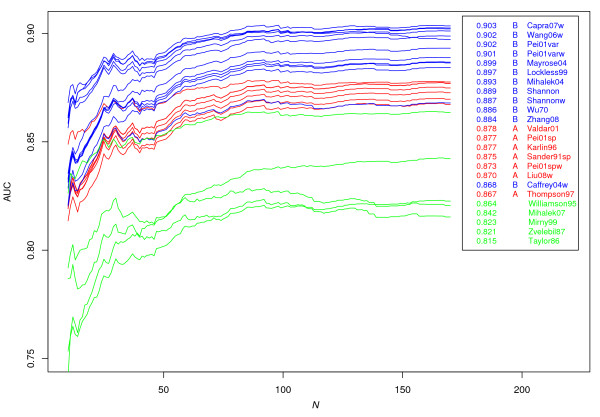
**Performance evaluation**. Performance evaluation of catalytic site prediction. Lines show the performance measured by the AUC measure for subsets of the original dataset obtained by setting an upper limit on alignment size (ranging from 10 to 168 sequences in an alignment). The legend is sorted on score performance for the original dataset (equal to the right terminal values of the graph), and also shows the numerical AUC value for this case. Lines and score names are colored acccording to the clustering shown in Figure 1 as; red: cluster A, blue: cluster B, green: other scores.

We evaluate the performance of scores in predicting catalytic sites, the result of which is shown in Figure 2. The figure shows performance measured by AUC for subsets of the original dataset derived by removing alignments containing more than *N *sequences. As expected, all methods perform worse for alignments containing fewer sequences. The color coding in Figure 2 shows that performance generally follows the classification given by the clustering discussed above, and that scores in cluster B perform best, followed by scores in cluster A. An exception is however Caffrey04w, which performs worse than most scores in cluster A. This is a quite surprising result, which calls for further study.

The scores outside clusters A and B are the least successful among all scores in predicting catalytic sites. As discussed above, four of these scores (Williamson95, Mirny99, Zvelebil87 and Taylor86) define their own alphabets of amino acid groups. We could conclude above that the manually designed amino acid groups gave scores that did not correlate strongly with other scores, and we can conclude from Figure 2 that this also makes the scores perform worse in predicting catalytic sites. We should however point out that some of these scores were not designed with catalytic sites in mind. For example, Mirny99 was designed to detect sites in the core of protein structures and Williamson95 was designed to detect ligand binding sites within the hydrophobic environment of the cell membrane. Mihalek07 was evaluated on protein-protein interaction sites, but is seen to lack some performance on our dataset. We suggest that the argument posed by Mihalek *et al. *that rare mutations should imply an important meaning of the site, is actually a drawback of their method. The possibility that rare mutations also implies non-conservation cannot be excluded. Mihalek07 is also sensitive to smaller alignments sizes, and is seen in Figure 2 to lose performance steadily as alignment sizes decrease.

Scores considering deviation from a background distribution generally perform better. One example of this is Williamson95 (relative Shannon entropy) which performs better than Mirny99 (Shannon entropy). The advantage of relative scores can also be seen from the scores in cluster B, which contains five scores measuring deviation from a background (Capra07w, Wang06w, Pei01var(w) and Lockless99). These scores occupy five of the top six rankings shown in the legend of Figure 2. Interestingly, these five scores also show greater tolerance for small alignment sizes. This can be compared to Mayrose04 (Rate4site) which performs among the best scores for large alignment sizes, but loses performance for alignments containing less than about 50 sequences.

With one exception, the scores from cluster A are seen to perform similarly across the entire spectrum of alignment sizes. The exception is Valdar01, which performs better for smaller alignments and also outperforms all "nonrelative" scores from cluster B for small enough alignments. There is indeed one property that separates Valdar01 from the other scores in cluster A--the ranking of completely conserved sites. While all other scoring methods in cluster A give equal scores to complete conservation, Valdar01 gives a higher conservation score to amino acids that have a high "self-similarity" value according to the substitution matrix used. While this is not the same as a relative score discussed above, there are similarities in practice. As an example, we may consider the rarest amino acid Tryptophan which also is the amino acid with the highest self-similarity value according to BLOSUM62. It is reasonable that this difference between Valdar01 and other scores in cluster A is amplified for alignments containing fewer sequences, since there may be more completely conserved sites in such alignments.

## Discussion and Conclusions

It can be difficult to evaluate similarities among computational methods. A formal classification based on method formulation does not always agree with similarity in performance in the actual application. One way of evaluating similarities among any computational methods is the empirical approach taken in this study. This approach was also recently taken to evaluate similarities of feature selection methods in microarray analysis [5]. In this study, we can also see some differences between practical performance and method formulation. The scores defined as stereochemically sensitive entropy scores were not clustered together, but instead seen to behave quite differently. The von Neumann entropy in Caffrey04w performed similarly to the Shannon entropy, while none of Mirny99 and Williamson95 were clustered close to any other score.

One key factor for this empirical approach is the selection of data to which the methods are applied. We used the Catalytic Site Atlas (CSA) data for this study, but we also tried to evaluate the scoring methods on Balibase [6] and Homstrad [7], which are alignment benchmark datasets. On both these datasets we could regenerate cluster A, cluster B and the other scores (data not shown), just as for the CSA data. The internal topology for these clusters were however slightly different on each dataset. We could for example see that sequence weights made a larger contribution to the internal topologies in clusters A and B on the Balibase dataset, suggesting that this dataset contains more alignments where sequence weighting is important.

To illustrate the difference between clusters A and B, we used as a representative case the site in the CSA dataset showing the largest difference in ranking between scoring methods Karlin96 and Shannon. All scoring methods' ranking of this site are shown in Table 2, where the scores have been normalized so that 1.0 implies that the site is judged to be the most conserved site in the alignment. This site has an amino acid profile containing 40% V and approximately 20% each of L, I and M. These are all hydrophobic amino acids which are deemed similar in BLOSUM62, and we can see that the scores from cluster A consistently give higher ranking than any method from cluster B. We can also see that Mirny99 judges this to be a completely conserved site, which is clear from the definition of Mirny99 where V, L, I and M are explicitly considered identical. For a conservation measure like the Shannon entropy, however, this site is far from conserved, since the most prevalent amino acid occupies only 40% of the amino acid profile.

**Table 2:** Example of ranking of a site

Mirny99		1.00	Caffrey04w	B	0.36
Thompson97	A	0.71	Zhang08	B	0.23
Zvelebil87		0.68	Mayrose04	B	0.20
Mihalek07		0.65	Shannonw	B	0.16
Liu08w	A	0.65	Pei01varw	B	0.16
Sander91sp	A	0.63	Wu70	B	0.15
Pei01sp	A	0.63	Shannon	B	0.15
Pei01spw	A	0.63	Pei01var	B	0.13
Karlin96	A	0.63	Wang06w	B	0.11
Taylor86		0.58	Lockless99	B	0.10
Williamson95		0.52	Capra07w	B	0.09
Valdar01	A	0.49	Mihalek04	B	0.08

Normalized rankings of alignment site with index 188 in alignment 1k32_A. The alignment site has the amino acid profile V: 41%, L: 22%, M: 22%, I: 15%.

This obvious difference in behaviour between clusters A and B highlights the question of how much attention should be given to "conservative mutations". The site depicted in Table 2 does indeed show a strong conservation of hydrophobicity. We can see that the scores in cluster B do not perform well in discovering this site, and a score that takes amino acid similarities into consideration would be preferred. From our performance evaluation we may conclude that conservative mutations should however not be considered when detecting catalytic sites. Inspection of the dataset gives that 60% of all catalytic sites are completely conservered, and 79% of all catalytic sites have an amino acid profile consisting of at least 90% of one specific amino acid. The interpretation of this is that conservative mutations are not tolerated in catalytic sites, and those scores that do consider amino acid similarities give false positives such as the one depicted in Table 2.

However, the conservation of features such as hydrophobicity may be a fruitful feature for other uses of conservation scores. For example in structural studies, it may be very useful to be able to discover such sites. Indeed, the Mirny99 score was developed for detecting the core of protein structures. We may finally notice that Caffrey04w gives a ranking of the site in Table 2 that is somewhere between clusters A and B. This might be expected since Caffrey04w is an entropy just like Shannon, but uses a substitution matrix like the scores in cluster A. However, as shown in Figure 2, the performance of Caffrey04w is not between clusters A and B, but instead below most scores from cluster A. We examine this score further and suggest an improvement in a separate study [8].

In summary--the biggest effect on the evaluation of an alignment site is the choice of whether amino acid similarities should be considered or not. We have concluded that, regarding catalytic sites, there is no benefit to be gained from considering amino acid similarities since it introduces false positives. The second biggest effect is the inclusion of background information. We could see that scores comparing the site with an expected background distribution perform better in predicting catalytic sites.

## Methods

### Scoring methods

We evaluate 25 scoring methods, all of which are shown in Table 3, with explanation of notations in Table 4. As far as possible, we present the scores following the same categorization as Valdar [1]. As can be seen in Table 3, several scores use a probability distribution *p_k _*for the amino acids at alignment site *k*. This distribution is easiest estimated by the observed frequencies of amino acids, but several methods add sequence weighting to solve taxonomic bias in the estimation of *p_k_*. The scores marked with "w" as suffix in the score name in Table 3 use sequence weighting by the Henikoff-Henikoff method [9].

**Table 3:** Scoring methods at an alignment site *k*

Symbol frequency	S	Wu70	*d_k_*/*p_k_*(α_0_(*k*))
	I	Lockless99	
	S	Pei01var(w)	
Stereochemical properties	I	Taylor86	min|{*T_j_*:*A_k _*⊆ *T_j_*}|, *j *= 1,...,61
	I	Zvelebil87	0.9 - 0.1*n_dis_*

Symbol entropy	S	Sander91	*S*_20_(*p_k_*)
	S	Shenkin91	
	S	Gerstein95	*S*_2_(*p_k_*) - *S*_2_(*p*)
	I	Wang06w	*R*(*p_k_*, *q*)
	I	Capra07w	(*R*(*p_k_*, *r*) + *R*(*q*, *r*))/2, *r *= (*p_k _*+ *q*)/2

Stereochemically sensitive entropy	S	Mirny99	*S_e_*(*p_k_*), *AA *= {AVLIMC, FWYH, STNQ, KR, DE, GP}
	S	Williamson95	*R*(*p_k_*, *p*), *AA *= {VLIM, FWY, ST, NQ, HKR, DE, AG, P, C}
	S	Caffrey04w	*V*(*p_k_*)

Substitution matrix	S	Sander91sp	
	I	Karlin96	
	S	Valdar01	
	S	Pei01sp(w)	
	I	Thompson97	
	I	Mihalek07	
	I	Liu08w	

Phylogeny	S	Mihalek04	
	I	Zhang08	
	D	Mayrose04	Rate4Site

Methods labeled as **S**: available in SEALA package at http://github.com/fredrikj, **I**: implemented for this study and available at http://github.com/fredrikj/bioruby, **D**: downloaded from the Rate4Site website http://consurf.tau.ac.il. Scores ending with "w" use sequence weighting by Henikoff and Henikoff [9]. Scores ending with "(w)" are used both with and without weights. Explanation of notations are given in Table 4, and further details can be found in the main text.

**Table 4:** Notations used in Table 3

*N*	No. of sequences in alignment.
*A_ik_*	The amino acid in sequence *i *at alignment site *k*.
*d*(*A_i_, A_j_*)	Sequence distance in percent.
*p_k_*	Probability estimated from site *k*.
*p*	Probability estimated from alignment.
*q*	Probability estimated from database.

*S_b_*(*k*)	
*R*(*p_k_, p*)	
*V *(*p_k_*)	-*Tr*(ω log_20 _ω), *Tr*(ω) = 1 ω = diag(*p_k_*(α_1_), ⋯, *p_k_*(α_20_)) × *M_f_*

*n_k_*	No. of occurences in site *k*.
*n*	No. of average occurences in a site.

*α_0_*(*k*)	Most common amino acid at *k*.
*d_k_*	No. of different amino acids at *k*.

*M*	The BLOSUM62 matrix, containing log-odds ratios (*blosum62.bla*).

*M_f_*	The BLOSUM62 matrix of frequencies (*blosum62.qij *).

*M_V_*	

*M_K_*	

*M_M_*	*M_f _*normalized such that each row and column approx. sums to 1.

*M_L_*	*M *normalized such that *M_L_*(α, α) = 10; 2 ≤ *M_L _*(α, β) ≤ 10

Pei *et al. *[10] suggested scores both with and without sequence weights and we include both alternatives in our analysis. Some other scores do not estimate *p_k _*but nevertheless have sequence weighting inherent in their definitions (Sander91sp, Valdar01).

Programs of 24 scoring methods were implemented by ourselves and out of these, 14 methods are available in the SEALA package, while 10 can be downloaded from the author's website. The Rate4Site method can be downloaded from its developers' website [11]. Table 3 shows the availability of methods.

#### Symbol frequency scores

Wu and Kabat [12] introduced the first widely accepted variation score to evaluate the variability of antigen recognition sites on antibodies. The score is simply the number of different amino acids at the site divided by the frequency of the site's most common amino acid. Lockless and Ranganathan [13] introduced a score that measures how amino acids found at the site deviate from the average distribution in the alignment. This is done by modelling occurences as binomial probabilities and calculating a root-squared sum of log-odds ratios for amino acids. Pei and Grishin [10] introduced a similar score, that takes a more straightforward approach of comparing distributions directly. Their score is a root-squared sum of distribution differences.

#### Stereochemical property scores

Some scores consider only the stereochemical properties at alignment sites, disregarding the exact identity of amino acids. Taylor [14] introduced a score in which 61 sets *T*_1_, ⋯, *T*_61 _were created, where each set contains amino acids sharing a stereochemical property. For example, the smallest set is *T*_4 _= {*D*, *E*} containing the negative amino acids Asp and Glu. The sets are not disjoint, and *T*_4 _is a subset of larger sets. The score is defined as the size of the smallest set *T_j _*that contains all amino acids at the alignment site. It is an integer between 2 and 20, and a smaller score indicates a higher conservation.

Zvelebil *et al. *[15] introduced a score that counts stereochemical dissimilarities occuring at a site. If only one amino acid is present at the site the score is set to 1, otherwise it is calculated according to the formula given in Table 3. This is done by considering ten stereochemical binary properties, and *n_dis _*is the number of properties that are found to vary at the site.

#### Symbol entropy scores

The symbol entropy scores use either the Shannon entropy *S *[16] or the relative entropy *R *(also known as Kullback-Leibler divergence), both of whose definitions are shown in Table 4. Sander and Schneider [17], Shenkin *et al.*[18], and Gerstein and Altman [19] all used a variant of Shannon entropy in their scores. Relative entropy measures the deviation of a probability distribution from a background distribution and was used by Wang and Samudrala [20], where they used the background distribution from the BLOSUM62 matrix. The rationale behind this score is that rare amino acids should get a higher score if they are conserved. A further development on this theme was done by Capra and Singh [21] who used the Jensen-Shannon divergence. This measure is very similar to relative entropy, with the advantage of being symmetric and limited between 0 and 1.

#### Stereochemically sensitive entropy scores

Entropy scores have also been adjusted to account for the stereochemical properties of amino acids. Mirny and Schakhnovich [22] did this by replacing the original alphabet of 20 amino acids by a smaller alphabet consisting of six amino acid groups. Williamson [23] developed a similar score, using a slightly different amino acid alphabet and using relative entropy with the distribution in the whole alignment as background. More recently, the von Neumann entropy (shown as *V *(*p_k_*) in Table 4) was introduced by Caffrey *et al. *[24]. This entropy was originally developed for quantum mechanics, and has been adapted to bioinformatics to account for amino acid similarities.

#### Substitution matrix scores

Substitution matrix scores do not consider residues by themselves, but rather consider the mutations that have occured. Sander and Schneider [17] introduced a sum-of-pairs score that sets a weight for each pair, depending on the sequence similarity. They use a scoring matrix where all values in the diagonal are one, which has the effect of giving the same score for any amino acid that is conserved. Karlin and Brocchieri [25] used an almost identical score, that only differed in that the score Karlin96 did not use sequence weighting. Valdar and Thornton [26] also used a similar score, but instead set individual sequence weights using a method from [27]. They normalize the substitution matrix *M *so that their matrix *M_V _*takes values in the range [0; 1] as shown in Table 4. Pei and Grishin [10] introduced a score that uses the same matrix as Sander91 and Karlin96, but sums over the alphabet of amino acids rather than the sequences in the alignment.

A vectorial view was proposed by Thompson *et al. *[28] in which one vector per sequence is defined for an alignment site *k*. The vectors are defined in the space of amino acids using a substitution matrix. A mean vector is then calculated for the site and the final score is the average euclidean distance to the mean vector. Mihalek *et al. *[4] developed a score that resembles relative entropy, considering amino acid pairs as the unit to measure. It counts the number of unordered pairs of amino acids *n_k_*(*α*, *β*) to estimate the probability *p_k_*(*α*, *β*). It then assumes a background distribution created by a substitution matrix normalized so that each row and column approximately sum to one (it is not possible to do exactly and keep a symmetric matrix). It is a score that rewards mutations (or lack of mutations) that deviate from this background. Liu and Guo [29] introduced a score that focuses on the most common amino acid (*α*_0_) in each site, and sums over comparisons of each amino acid with *α*_0_.

#### Phylogeny scores

Mihalek *et al. *[30] developed the real-valued evolutionary trace method, which is a score to quantitatively measure evolutionary trace [31]. They do this by constructing a phylogenetic tree using the UPGMA method, and summing entropies on the groups created by cutting the tree at different nodes.

Zhang *et al. *[32] later introduced an extension of Mihalek04 in which the Shannon entropy was replaced by the von Neumann entropy. Mayrose *et al. *[33] developed Rate4Site, a computationally demanding method that estimates evolutionary rate (thus conservation). It does this by constructing a phylogenetic tree and inferring an evolutionary rate for each site using a Bayesian inference scheme, given a statistical model for evolution. This score is the default method at the ConSurf web server [34], and is the successor of the original ConSurf method [35] which we do not include in this study.

#### Scores not included

Several scoring methods are not included in this study. For example, cumulative relative entropy [36] and quantitative evolutionary trace [37] are not included. They are designed to detect alignment sites involved in the functional difference between subsets of the aligned proteins. Both methods require information about the classification of aligned sequences into subsets corresponding to the functional difference, and we therefore do not include the methods in this study.

Integer-valued evolutionary trace and zoom methods are associated with real-valued evolutionary trace [30]. Integer-valued evolutionary trace was used to demonstrate the superiority of real-valued evolutionary trace, whereas zoom focuses on a single sequence. Therefore, the two scores are excluded from our analysis.

We do not include 3 D cluster analysis [38], which calculates the score at an alignment site by considering the site itself along with alignment sites of spatially close residues. We do not consider structure in this study, and do not include this score.

Valdar [1] suggested a score that is a multiplication of three scores--Shannon entropy, a score similar to the score by Thompson *et al. *[28] and a gap penalty. The score is highly dependent on the weight for each factor in this multiplication, so we do not include it in this study. It also includes a gap penalty factor and we do not evaluate score performances on gaps in this study.

### Score evaluation

We evaluate scores on a subset of the Catalytic Site Atlas (CSA) [2], compiled in [21]. We analyze all alignment sites without gaps or undecided amino acids (B, X, Z) which leaves us with a dataset consisting of 455 alignments with a total of 89446 sites, of which 1149 are catalytic sites. Hence, the scores' evaluations of gaps is not included in this study. Any score could however be extended with a gap penalty to handle this.

The purpose of a conservation or variation score is generally to compare sites within an alignment, and we analyze specifically this property of the scoring methods. In order to do this, we negate scores that give high scores for high variability, so that all scores can be viewed as conservation scores. Further, when comparing sites within an alignment, the scores Sander91, Shenkin91 and Gerstein95 give equal comparisons since they are all variations of a Shannon entropy. We therefore evaluate them together as a score which we name Shannon (which is identical to the Sander91 score). We also evaluate the score Shannonw as suggested by Pei *et al. *[10] which uses sequence weights for the estimation of *p_k_*.

We also show that a comparison of scoring methods other than within alignments is difficult, by evaluating how the scores depend on alignment size (the number of sequences in the alignment). We do this by, for each scoring method, calculating the Pearson correlation between alignment size and mean score on the alignment.

#### Cluster analysis

We perform hierarchical cluster analysis on the scoring methods, in order to see how scoring methods relate to each other. For this analysis we calculate a distance between every possible pair of scoring methods. We do this by calculating a correlation matrix *C*^(*i*) ^for each alignment *i*, where the element  is the correlation between methods *k *and *l *on sites in alignment *i*. We subsequently calculate the average correlation matrix  for all alignments (*m *= 455 which is the number of alignments). Hierarchical clustering is then done on the distance matrix . The symbol **1 **denotes a unit matrix, i.e. a matrix consisting only of ones. Since we are interested in how the scores compare sites, i.e. the ranking of sites, we use the Spearman's rank correlation which also solves possible problems with outliers and nonlinear correlations.

At each stage of the hierarchical clustering, the two closest clusters (created at earlier stages) or single units are merged into a new cluster. Above we defined the distance between units (the scoring methods) but we also have to define the distance betweeen clusters, i.e. we have to define a clustering method. We choose to mainly present average linking as clustering method, which means that clusters are joined based on the average distance between all inter-cluster pairs. Other possible methods include single linking and complete linking. In single linking the distance between two clusters is equal to the distance between the closest inter-cluster pair, while in complete linking it is equal to the distance between the most distant inter-cluster pair. Both these methods are vulnerable to outliers, since they merge clusters based only on local measures. Therefore, we mainly discuss the clustering of scoring methods based on the average linking method. For comparison, we however also perform hierarchical clustering using single and complete linking. We also perform a bootstrap procedure to determine the reliability of each node in the resulting dendrogram. One bootstrap sample *j *is obtained by randomly picking *m *alignments from the original dataset (where one alignment may be picked more than once) and calculating a distance matrix  in the same way as above. We obtain 1000 bootstrap samples in this way, and each node in the original dendrogram is labeled with the number of times (in percent) that an identical node is found in a bootstrap dendrogram (identical in the meaning of having the same set of leaves under it).

#### Performance evaluation

We evaluate the performances of scoring methods in predicting catalytic sites in the alignments. We do this by calculating the receiver operator characteristic (ROC) curve for each score and alignment. We subsequently calculate the area under each ROC curve (AUC), and calculate an average AUC score on all alignments for each score. We also evaluate how performance depends on the size of alignment, by setting upper bounds on alignment size and evaluating AUC scores only for alignments with a size smaller than that.

## Authors' contributions

HT conceived the study. FJ implemented methods and performed the analysis. All authors read and approved the final manuscript.

## Supplementary Material

Additional file 1 **Single linking hierarchical clustering**. Dendrogram created using single linking. Click here for file

Additional file 2 **Complete linking hierarchical clustering**. Dendrogram created using complete linking. Click here for file
