# Supplementary figures and images for: A comparative study of conservation and variation scores
Source: BMC Bioinformatics. 2010 Jul 21;11:388. doi: 10.1186/1471-2105-11-388 (PMC2920274; doi:10.1186/1471-2105-11-388)

# Single linking

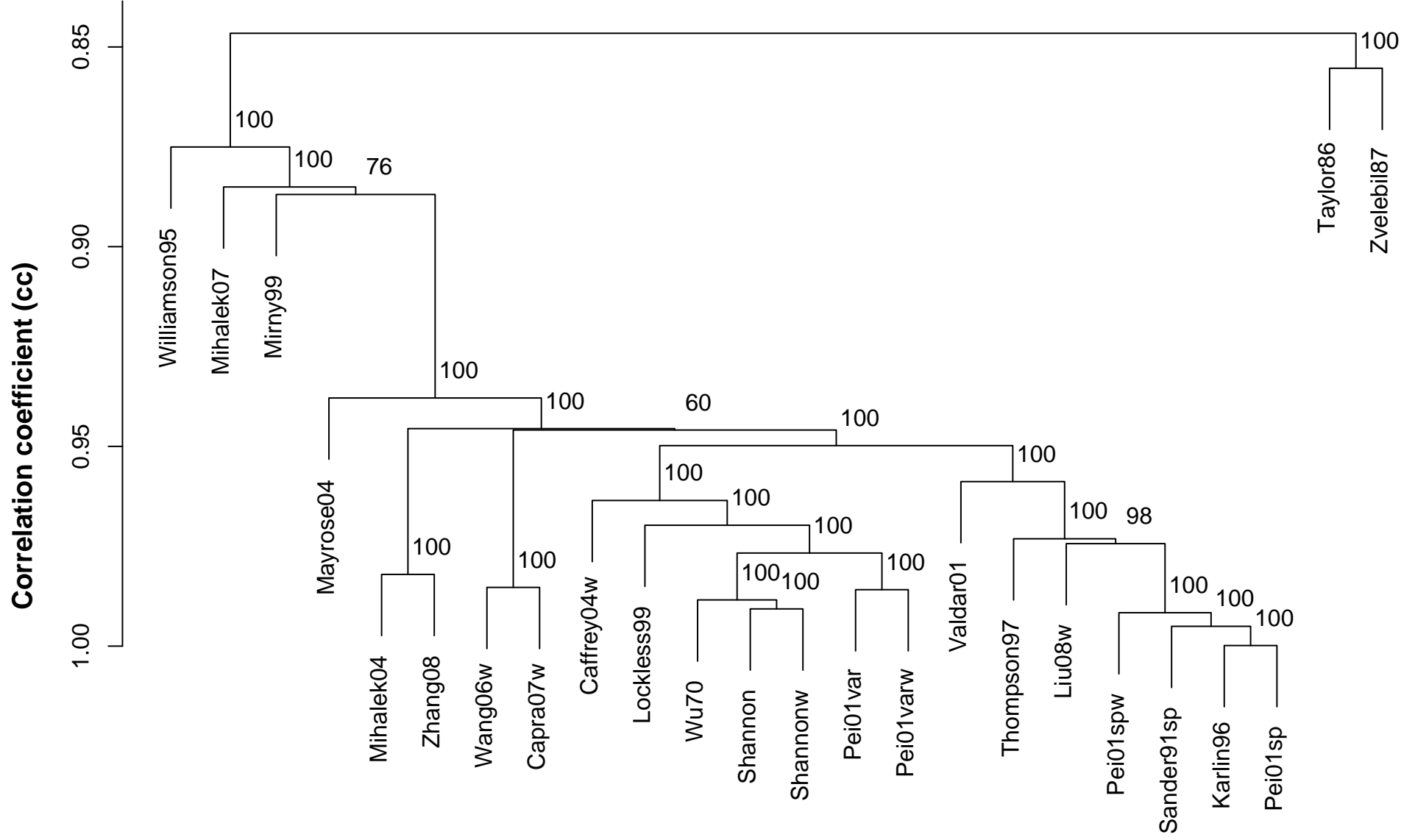

Supplement: Additional file 1 — Single linking hierarchical clustering. Dendrogram created using single linking. [file 1471-2105-11-388-S1.PDF]

## Complete linking

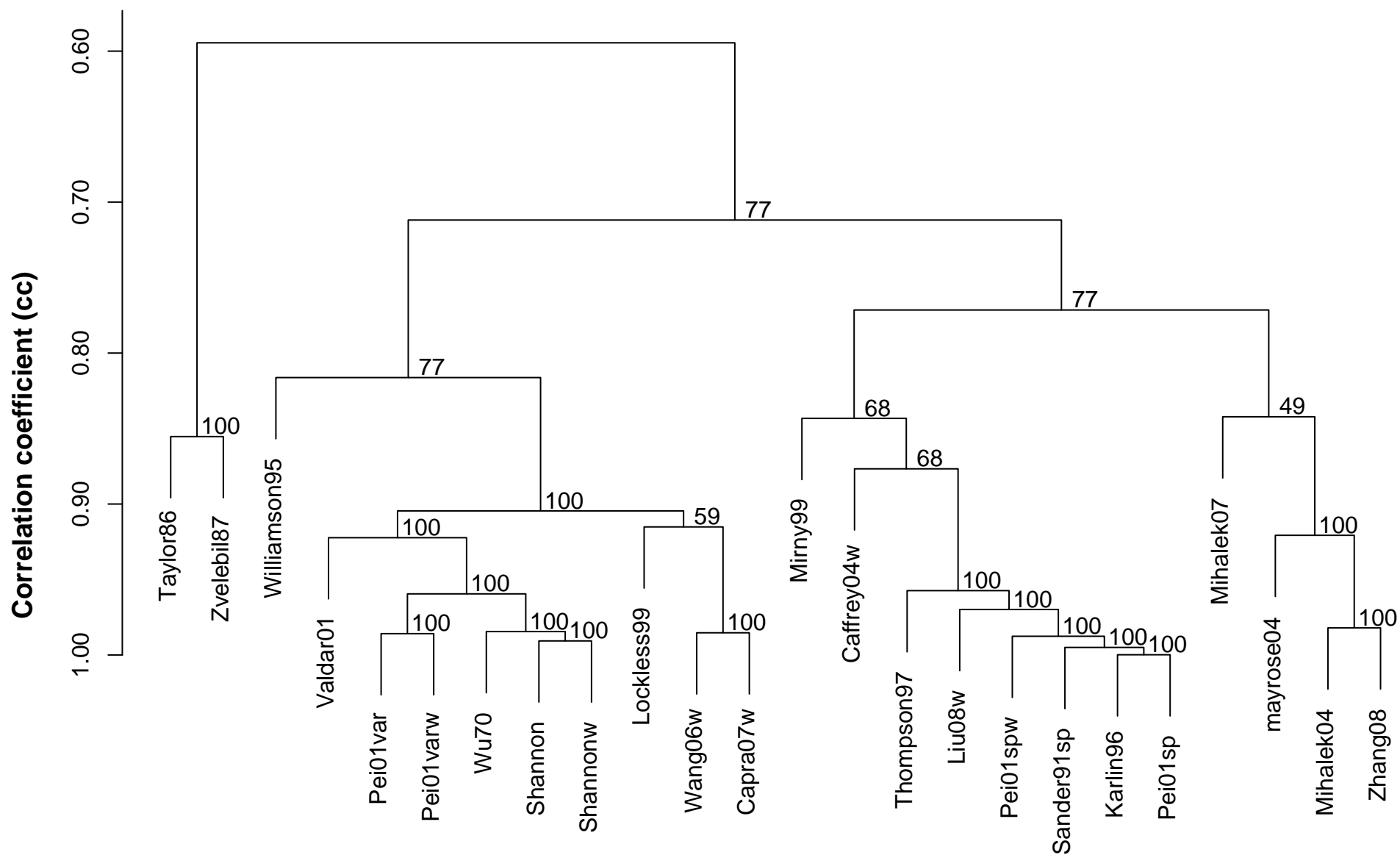

Supplement: Additional file 2 — Complete linking hierarchical clustering. Dendrogram created using complete linking. [file 1471-2105-11-388-S2.PDF]
